# Lysogeny in *Streptococcus pneumoniae*

**DOI:** 10.3390/microorganisms8101546

**Published:** 2020-10-07

**Authors:** Geneviève Garriss, Birgitta Henriques-Normark

**Affiliations:** 1 Department of Microbiology, Tumor and Cell Biology, Karolinska Institutet, 171 77 Stockholm, Sweden; 2 Clinical Microbiology, Karolinska University Hospital, Bioclinicum, 171 76 Stockholm, Sweden; 3 Lee Kong Chian School of Medicine (LKC) and Singapore Centre on Environmental Life Sciences Engineering (SCELSE), Nanyang Technological University, Singapore 639798, Singapore

**Keywords:** temperate bacteriophages, *Streptococcus pneumoniae*, lysogeny, phage phylogeny, phage integration, PblB

## Abstract

Bacterial viruses, or bacteriophages, are major contributors to the evolution, pathogenesis and overall biology of their host bacteria. During their life cycle, temperate bacteriophages form stable associations with their host by integrating into the chromosome, a process called lysogeny. Isolates of the human pathogen *Streptococcus pneumoniae* are frequently lysogenic, and genomic studies have allowed the classification of these phages into distinct phylogenetic groups. Here, we review the recent advances in the characterization of temperate pneumococcal phages, with a focus on their genetic features and chromosomal integration loci. We also discuss the contribution of phages, and specific phage-encoded features, to colonization and virulence. Finally, we discuss interesting research perspectives in this field.

## 1. Introduction

Bacteriophages, or, simply, phages, are the most common biological entity on Earth. These bacterial viruses are found in every ecological system, playing profound roles in many aspects of life from bacterial pathogenesis to the global ecology of our planet [1,2]. Historically, phages are immensely significant, as they enabled the early studies that laid the cornerstones of modern genetics and molecular biology. Due to their simplicity, they were instrumental in understanding fundamental principles in biology, such as the nature of the gene and the role of mRNA in translation (reviewed in [3,4]). The first genomes to be ever sequenced were those of RNA and DNA phages MS2 and ϕX174 [5,6].

**Figure 1:**
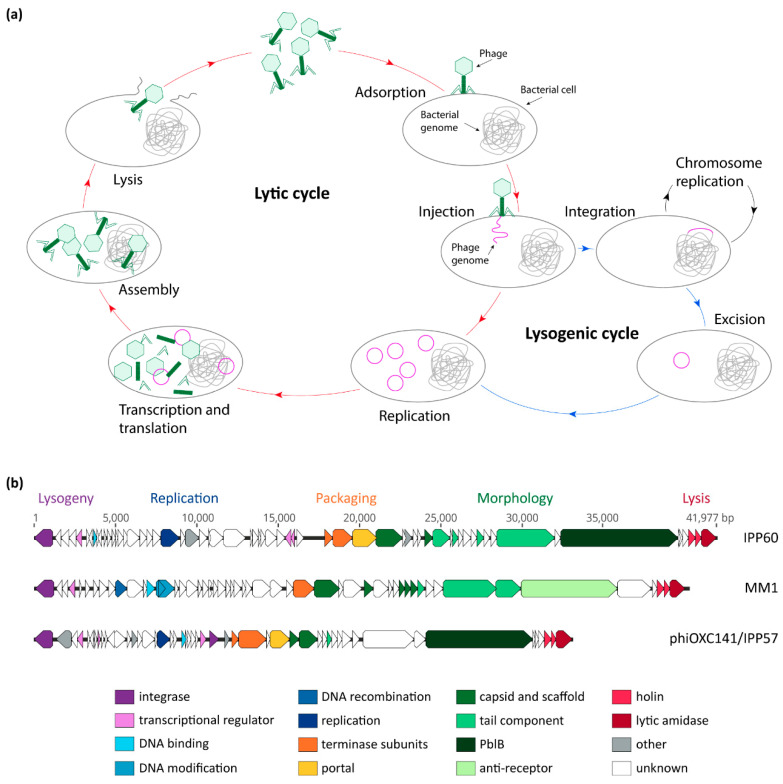
Bacteriophage life cycles and genetic modules. (**a**) General model of phage lytic and lysogenic cycles; (**b**) comparison of the sequences of three pneumococcal temperate phages: IPP60 (KY065496.1), MM1 (NC_003050.2), and phiOXC141/IPP57 (KY065494.1). Annotated open-reading frames are represented by arrows that point in the direction of transcription, and are color-coded according to their putative function.

Phages are obligate intracellular parasites; they must infect a host in order to replicate their genetic material, multiply, and disseminate. Phages are categorized into two groups, based on their life cycle: (i) virulent phages, which can only perform a lytic cycle; and (ii) temperate phages, which can undergo both lysogenic and lytic cycles [4]. In the first steps of its life cycle, the phage adsorbs to receptors located on the surface of its bacterial host cell and injects its genome into the cell (Figure 1a). Virulent phages then undergo the lytic cycle, during which their genetic material is replicated, transcribed, and translated, and new virions assembled. Temperate phages can, however, establish themselves as part of their host’s genome—along which they are replicated during normal cell division—typically by integrating into the chromosome. In its integrated form, the phage is called a prophage, and a bacterium harboring a prophage is a lysogen. During lysogeny, the prophage is replicated, along with the host chromosome, and passed on to daughter cells. This association will continue until stress conditions, such as exposure to DNA-damaging agents, induce the prophage to excise from the chromosome and enter the lytic cycle. Newly-formed phage particles are then released through lysis of the host cell, and can infect another host bacterium.

The consequences of lysogeny for the bacterial host are diverse, and temperate bacteriophages undeniably play an immense role in bacterial genome evolution. Besides mobilizing their own genetic material, temperate phages can promote the horizontal transfer of host genes through generalized and specialized transduction, and act as helper elements for the transfer of phage-related chromosomal islands (PRCIs) [4,7]. They can also contribute genes that will be expressed during lysogeny, or by a lytic phage subpopulation, in a process termed lysogenic conversion [8]. These genes can be potent toxins which significantly contribute to pathogenesis, such as the shiga toxins of *Escherichia coli* O157:H7, the diphtheria toxin of *Corynebacterium diphteriae,* and the cholera toxin of *Vibrio cholerae* [9,10,11]. Finally, some temperate phages that integrate within bacterial coding sequences can act as genetic switches, through controlled excision and integration reactions, whereby allowing the timely expression of their integration target. This phenomenon is termed active lysogeny, and has been adeptly reviewed by Feiner et al. [8].

Bacteriophages infecting the human pathogen *Streptococcus pneumoniae* (*Spn*) were first reported in 1975 by two independent groups, some 60 years after the separate discoveries of phages by F. d’Hérelle and F. W. Twort [12,13,14,15]. There was an almost immediate interest in understanding the distribution of bacteriophages in clinical isolates of *Spn,* and if they contribute to pneumococcal diseases [16,17]. Most pneumococcal phages reported to date are temperate, but despite being less prevalent, virulent phages such as Dp-1 and Cp-1 have been useful for understanding pneumococcal and phage cell wall hydrolases (reviewed in detail by López and García [18]). Phages have been known to be highly prevalent in pneumococcal genomes since the late 1970s, with early estimates that up to 76% of pneumococcal isolates carry temperate phages [16,17,19]. Refined genomic analyses have confirmed the widespread distribution of prophages in pneumococcal genomes [20,21,22]. This review focuses on the recent advances on the genomic and functional characterization of pneumococcal temperate phages and their contribution to pneumococcal pathogenesis and genome evolution, and highlights some of the knowledge gaps and interesting avenues for future research.

## 2. Pneumococcal Bacteriophage Genetic Modules

The first pneumococcal temperate phage to be sequenced was MM1, in 2003 [23]. Since then, thousands of pneumococcal isolates—and their integrated prophages—have been sequenced, providing a wealth of genomic data to study phage genetics and genomics, as well as phage–host interaction and epidemiology. A recent study encompassing 482 *Spn* genomes found that 100% of them contain at least a few phage genes, and the total phage content of a given strain could account for as much as 6% of the genome size [20]. In total, 45% of genomes were found to carry at least one putatively full-length phage, and 13% of genomes were found to carry more than one phage [20]. As of today, remarkably few studies have been dedicated to elucidating the regulation mechanisms of *Spn* phages, and the role that most of the phage gene products play in the context of the phage life cycle remains unknown [24,25]. Nonetheless, based on analysis of their predicted coding sequences, pneumococcal phages are organized in genetic modules which encompass all functions required for the phage life cycle (lysogeny, replication, packaging, morphology, and host cell lysis), and the order of these modules is conserved between phages (Figure 1b) [20,21,23,26,27].

The lysogeny module, located at the 5′ of the integrated phage, includes genes involved in integration/excision from the chromosome (described in detail below) and transcriptional regulation. Lysogeny by phage lambda, the paradigm of temperate phages, is maintained by the action of the phage CI protein, which represses the two divergently oriented early lytic promoters P_R_ and P_L_ (reviewed in [28,29]). When DNA damages occur, the bacterial RecA protein becomes activated by the presence of intracellular single-stranded DNA and catalyzes the auto-catalytic cleavage of CI, leading to de-repression of the phage promoters. Phage MM1 was shown to harbor an analog of CI which represses elongation of the transcripts initiated from two divergently oriented promoters located in the lysogenic module [25]. In many bacterial species, including *E. coli*, the natural host of phage lambda, activated RecA also catalyzes autoproteolysis of the host LexA repressor, triggering induction of the SOS response regulon (extensively reviewed in [30]). With the exception of RecA, pneumococci lack the genetic components of the classical SOS response pathway. However, the competence for natural transformation regulon, which includes RecA, is thought to be the functional replacement of the SOS system, and is similarly induced by DNA-damaging agents [31]. In accordance with this idea, *Spn* temperate phages are also induced by DNA-damaging agents, such as mitomycin C (MMC), UV light, and fluoroquinolones, via a RecA-dependent mechanism [20,32,33,34,35].

Downstream of the lysogeny module is the replication module. None of the replication module genes have been functionally validated, however, homologs of proteins involved in DNA replication, binding, recombination, and methylation, and resolution of Holliday junctions, have been identified [20,23,26,32]. The packaging module, whose role is of packing the viral genome to the newly-formed phage heads (proheads), follows the replication module. Genome packaging is normally achieved by the small and large terminase subunits, in concert with the portal protein, which sits at the entrance of the prohead (reviewed in detail in [36]). One or two genes encoding terminase subunits, and one encoding a portal protein, are found in most pneumococcal phages studied [26].

The morphology module ensures production of the structural components of the phage particles, and is located downstream of the packaging module. All temperate pneumococcal phages observed, to this day, are double-stranded DNA tailed bacteriophages that belong to the *Siphoviridae* family [17,24,26,27,37,38]. Genes encoding the structural components of the phage particles have only been identified based on sequence similarities to phages from other bacterial species. The PblB protein encoded by a large number of pneumophages is thought to be the antireceptor, the protein responsible for mediating recognition of the host cell. No experimental evidence for this is available, however, studies in *Streptococcus mitis* have shown that the PblB protein encoded by temperate phage SM1 is a structural component of the phage tail [39]. In phages that lack PblB, such as MM1 and its related phages, *orf47* and its homologs are thought to encode the antireceptor [23]. No candidate has been so far proposed as the receptor for *Spn* temperate phage adsorption; however, the choline residues in the pneumococcal cell wall are required for infection with the virulent pneumococcal phage Dp-1, and PblB_SM1_ can bind choline residues when present in the extracellular environment [12,40,41]. Whether this is also the case for temperate pneumococcal phages is unknown. The presence of the polysaccharide capsule has been shown to inhibit phage infection with virulent phages under laboratory conditions, presumably by limiting accessibility of the receptor [42,43]. However, the ability of pneumococci to undergo phase-variation, which results in variations in the amount of capsule produced [44], provides a context in which phage infection would not be inhibited by the capsule at all times, and would contribute to allow the broad distribution of phages seen in capsulated pneumococcal isolates. The transparent phase, which is associated with a reduced capsule production, is linked to enhanced nasopharyngeal colonization [45,46], which could facilitate phage transmission via close cell-to-cell contacts.

The lysis module is located at the 3′ end of the prophage, and encompasses typically one or two holins and an *N*-acetylmuramoyl-L-alanine amidase (also referred to as endolysin, lytic amidase, or phage *lytA*) [20,26]. The activities of both holin and lytic amidase are required for phage-mediated lysis. Holin-induced permeabilization of the membrane allows the lytic amidase, which normally remains in the cytosol, to gain access to, and degrade, the cell wall [47]. Lytic amidases encoded by pneumococcal phages share a strong similarity with the chromosomally-encoded autolysin LytA, and also require the presence of choline in the cell wall for their hydrolytic activity [26,48,49,50]. LytA is, moreover, activated during phage-mediated cell lysis, and can complement the lack of the phage lytic amidase in the release of a functional phage progeny [51].

## 3. Phage Groups and Chromosomal Integration Loci

An initial comparative genomic analysis of ten pneumococcal temperate phage genomes delineated three phage phylogenetic clades [26]. A broader analysis indicates that most phages belong to these three major groups, with only a small number of divergent phages falling outside these clusters [20]. Phage groups 1, 2, and 3 correspond respectively to phage clusters B1–B4, C, and A, described by Brueggemann et al. [20], however, for the sake of simplicity, we will employ here the earlier names proposed by Romero et al. [26], as they were used for classifying phages in several subsequent publications [35,38,52,53]. Phages belonging to the same phylogenetic group share high sequence similarity in their packaging, morphology, and lysis modules, and are typically associated with one or two main integrase types [20,26]. Additional genes encoding replication, DNA binding, and hypothetical proteins are also group-specific. In contradiction with the theory of modular evolution of phages, pneumococcal phages do not appear to evolve through exchange of functional modules, and there is but little evidence for exchange of genes between phages of different groups [20,26,32,54].

**Figure 2:**
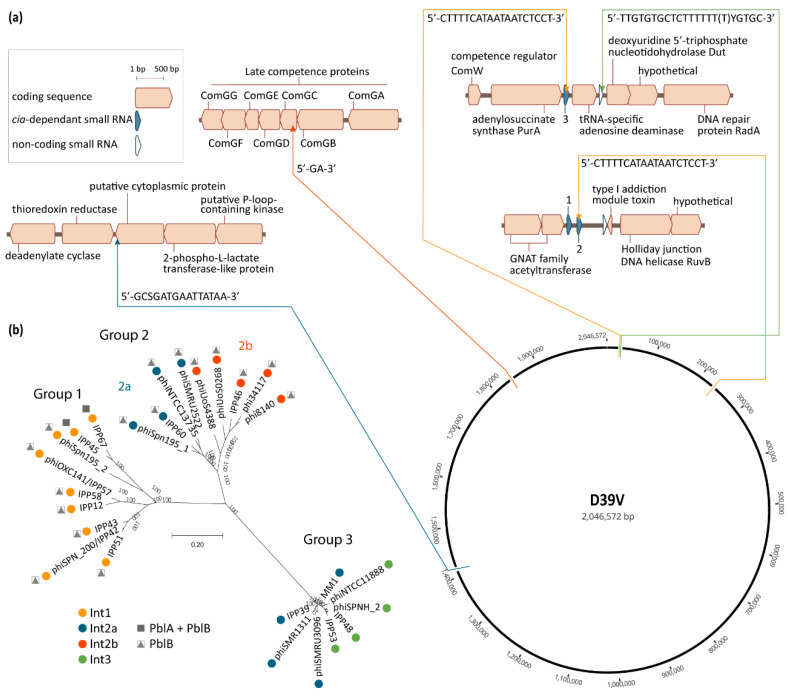
Pneumococcal temperate phage groups and their chromosomal integration loci. (**a**) Chromosomal integration sites of pneumococcal phages. Thin arrows indicate the precise locus of the phage attachment sites, in relation to their immediate genetic context and their location on the circular representation of the D39V (CP027540.1) genome. Direction of the arrows reflects the orientation of phage integration in the chromosome respective to the integrase being located at the 5′ end of the integrated phage, as is the convention. Color of the arrows and connecting lines refer to the integrase type in panel (b). The nucleotide sequence of each core attachment site is indicated at each integration locus, as previously defined by Romero et al. [26] (Int1) and Gindreau et al. [24] (Int2a, including modifications reflecting single nucleotide polymorphisms in the genomes used in panel (b)), or as determined by the authors by aligning the left and right phage–chromosome junctions with the corresponding chromosomal site in the genome of D39V, in the case of Int3 and Int2b; (**b**) phylogenetic tree of a selection of pneumococcal temperate phages for which the genetic context could be determined. The multiple alignment of phage genomes was performed using MUSCLE v.3.8 [60]. The phylogenetic tree was constructed using FastTree v.2.1.10 [61] and visualized in MEGA-X v10.1.8 [62]. Colored dots represent the integrase type according to the key, and refer to the similarly-colored arrows pointing to the integration sites in panel (a). All Int1-encoding phages depicted here, except for IPP67, integrate in csRNA3. The presence of genes encoding the putative virulence factors PblA and PblB is indicated. Accession numbers of the sequences used are listed in Appendix A, Table A1.

Pneumococcal phages are associated with four different integration sites located in a total of five chromosomal loci (Figure 2a) [20,21,26]. The positioning of these sites results in pneumococcal prophages being co-oriented with the bacterial replication fork, and the majority of their coding sequences oriented in the 5′–3′ direction on the leading strand template (Figure 2a). A similar preference has been observed for prophages in other bacterial species [55,56,57]. The site-specific recombination reaction leading to phage integration in the bacterial genome is mediated by the phage integrase through recognition and recombination of specific DNA sequences located on the bacterial and phage genomes [58]. These sequences or “attachment sites”, respectively named attB and attP, share a common core sequence where the crossover occurs. This core region is also found in the attL and attR sites which flank the integrated phage, as they are each composed of half of attB and half of attP. While recombination between attB and attP leads to phage integration, the inverse reaction—recombination between attL and attR—leads to excision of the phage from the chromosome. This latter reaction is carried out by the integrase, and is normally assisted by a recombination directionality factor (RDF), also called excisionase [59]. No excisionase has been, to this day, identified in pneumophages. Excisionases are reputedly difficult to identify, due to their small size and lack of sequence conservation, but often have a high predicted isoelectric point [59]. Different integrases recognize different attachment sites, thus leading to integration of the phages that encode them in different chromosomal locations [58].

Based on sequence similarity, four major integrase types are found in pneumococcal phages. Int1, Int2a, and Int3 belong to the tyrosine recombinase family, while Int2b belongs to the serine recombinase family. These integrases are each associated with specific core sites which can be found on each side of the integrated phage (attL and attR) and in the chromosome of non-lysogenic isolates (attB). All phages carrying Int1 fall within group 1, and conversely, almost all group 1 phages encode Int1, with the exception of IPP5, reported by Brueggemann et al. [20] (Figure 2b). The attB site associated with Int1 is found in three instances in the pneumococcal genome, namely within the sequence of the non-coding *cia*-dependent small RNAs csRNA3, csRNA2, and csRNA4 [26,32]. The majority of phages carrying Int1 appear to integrate within csRNA3, which is located between the genes encoding the adenylosuccinate synthase PurA and a tRNA-specific adenosine deaminase (Figure 2a). Some group 1 phages, such as IPP67, integrate in csRNA2 (Figure 2b). Experimental validation indicates that phage SpSL1, which encodes Int1, is capable of integrating in both csRNA3 and csRNA2, but that integration in csRNA2 occurs only in conjunction with integration of another copy of SpSL1 in csRNA3 [32]. Interestingly, the phage sequence located between the core recombination site and Int1 is nearly identical to the sequence of csRNA2, leading to reconstitution of the csRNA2 sequence following phage integration. Integration into csRNA3 leads to the formation of a likely functional chimeric csRNA composed of the upstream part of csRNA3 and the downstream part of csRNA2 [32]. While blastn analysis reveals that various pneumococcal genomes do harbor phages integrated within csRNA2, none appear to be found within csRNA4. Since phage integrases typically require additional sequence features other than the core site [58], it is possible that differences in the sequence surrounding the core attB site in csRNA4 prevent phage integration at this locus.

Group 2 phages have been further subdivided into two subgroups, based on the integrase they encode, Int2a or Int2b, and phages carrying each integrase appear to be phylogenetically more related (Figure 2b and [53]). The core attB site of phages encoding Int2a is located within the 3′ end of a gene encoding a putative cytoplasmic protein (SPV_1394) annotated as DNA-binding protein WhiA in some genomes, and phage integration at this locus reconstitutes the normal 3′ end of the coding sequence [24,26]. Int2a is also encoded by some group 3 phages, such as MM1 (Figure 2b) [20,24,26]. Int2a is the only pneumococcal phage integrase for which there is experimental evidence of its role in phage integration and excision [24,63]. Phages encoding Int2b are found integrated within the gene encoding the late competence protein ComGC [53], between nucleotides 58 and 59 (GG, unpublished data). Int2b is the only pneumococcal phage integrase described today which is associated with disruption of a coding sequence. ComGC is the major pilin of the competence pilus [64], and in accordance with its essential role in competence, isolates carrying a phage integrated within *comGC* are not transformable [53].

Group 3 phages encode either Int2a or Int3, with no apparent intragroup clustering related to integrase type. The core attB site associated with Int3 is located between the coding sequences of a tRNA-specific adenosine deaminase and the deoxyuridine 5′-triphosphate nucleotidohydrolase Dut (Figure 2b) [26]. A more detailed analysis reveals that the core site partially overlaps the 3′ end of the ncRNA SPV_0026, but that phage integration does not disrupt its sequence (GG, unpublished data). The few phages described that do not fall into the three main groups described here encode either Int2a or unique integrases [20].

## 4. Polylysogeny

The presence of more than one prophage per genome, or polylysogeny, has been reported in various studies, and appears to be a relatively frequent feature of *S. pneumoniae* [20,38,52,65]. A detailed comparison of phages found in polylysogenic isolates has not been performed, however, it appears to occur with phages that belong to distinct phylogenetic groups and with distinct integration sites [20,38,52,65]. This might reflect inability of phages to integrate in tandem in the same attB site, or the activation of phage superinfection resistance mechanisms. Multiple mechanisms, such a receptor modification or removal, transcription or replication blockage, and inhibition of phage genome injection, have been described, and are adopted by resident phages to block the entry of a second related phage [66,67,68].

Despite the easy and relatively cheap access to whole-genome sequencing, assembly of sequencing data is confounded by the nature of pneumococcal phages themselves, such as the presence of repeated domains within the *pblB* variants encoded by many phages and homology with chromosomal genes such as between the host *lytA* gene and phage lytic amidases [20,48,49,65]. Mobile genetic elements (MGEs) are notorious for creating genome assembly problems, with contigs often ending within MGE sequences [69]. This is particularly the case with short-read sequencing technologies where repeats can be longer than the reads generated [70]. Polylysogeny further complicates this issue, due to similarities between genes harbored by different phages present in the same genome, even when they belong to different phylogenetic groups. *pblB* variants and lytic amidases are, again, good examples here, as they are encoded by many (*pblB*), or virtually all (lytic amidase) pneumococcal phages. In addition, spontaneous phage induction appears to be common among pneumococcal phages [19,63,71], and the co-existence of integrated and excised forms poses a problem for the reliable assembly of phage genomes (unpublished observations).

## 5. The Phage and the Host: Contribution to Virulence and Colonization

Soon after the discovery of phages infecting the pneumococcus, there was an interest in understanding if they play a role in pathogenesis [17]. Interestingly, RNA sequencing data indicates that phage gene expression is higher during planktonic growth—a condition akin to bacteremia—compared to during growth in a biofilm [72]. Phages have been described in isolates of globally-circulating successful clones, such as PMEN1 (Spain^23F^-1) and PMEN3 (Spain^9V^-3), and a number of studies have investigated the possible association of prophages in specific predominant serotypes or lineages [20,52,65,73,74,75,76,77]. Some phages were found to be strongly associated with specific clonal lineages, such as phage MM1 with the PMEN1 lineage, and some phages have been found to persist over decades (more than 60 years in the case of IPP34, a group 2a phage) [20]. However, large-scale pneumococcal population genomic studies have also shown that phages are not as stably associated with pneumococcal lineages as other types of mobile genetic elements, such as integrating conjugative elements, and that prophage content is dynamic and transient, even within individual carriage episodes [21,22,53]. Together with intragenomic recombination, phage transmission emerges as the main contributor to short-term evolution within clonal populations [21]. Perhaps unsurprisingly, phage remnants appear more stably associated with specific lineages, suggesting that degradation of phage sequences is biased towards the conservation of specific features which provide an advantage to the host [21]. This is also the case with phage-related chromosomal islands (PRCIs, see below), and conservation of some PRCIs across decades with virtually no sequence divergence supports the idea that they also contribute specific advantages to their host [21,72].

The presence of homologs of the PblA and PblB proteins highlights possible roles for temperate phages in the virulence of *S. pneumoniae*. Indeed, a genome-wide association study found that the presence of *pblB* was an independent and positive determinant of 30-day mortality in bacteremic patients [34]. Initially described in temperate phage SM1 from *S. mitis*, PblA and PblB were shown to mediate binding to platelets and contribute significantly to the virulence of *S. mitis* in a rabbit endocarditis model [39,41,78]. Experimental data indicate that PblA_SM1_ and PblB_SM1_ can be both cell-wall associated and structural components of the SM1 phage tail [39]. The mechanism through which these proteins are selectively targeted to the cell wall or phage particles is unknown. However, the phage-encoded holin and lysin are required for release of PblA and PblB in the extracellular milieu, upon which they can bind the surface of intact cells via choline residues contained in the cell wall [41]. All pneumococcal phages belonging to groups 1 and 2 encode variants of PblB, and a subset of group 1 phages (cluster B2 in [20]) encode in addition PblA (Figure 2b) [20,26]. None of the group 3 phages encode either of these proteins, nor do the few phages that cluster outside the three main groups [20]. In agreement with their role for the prophage itself, expression of PblA and PblB is induced by DNA-damaging agents, such as mitomycin C and fluoroquinolones, in both *S. pneumoniae* and *S. mitis* [20,34,39].

The PblA protein encoded by *Spn* phages has not been investigated to date, but its sequence is highly conserved [20] and shares nearly 70% identity with PblA_SM1_. The pneumococcal phage PblB proteins display a much higher level of diversity, and the average sequence identity of *pblB* genes is less than 65% [20]. Variants of PblB encoded by pneumococcal phages have been shown to promote adhesion by binding to galactose in the glycoconjugates present on the surface of lung epithelial cells, to stimulate colonization in vivo [79], and to enhance platelet activation [34]. Deletion of a PblB-encoding phage associated with hypervirulent isolates of serotype 1 was shown to increase the number of circulating platelets and to cause a significant decrease in the ability to adhere to lung epithelial cells and nasopharyngeal cells, as well as to form a biofilm on fixed lung epithelial cells [75,76]. In vivo experiments show that deletion of this prophage leads to a reduction in the number of bacteria in the lungs, but this reduction is rather modest, and restricted to the early stages of infection (12 and 24 h) [76]. In contrast, the presence of phage Spn1, which also encodes PblB, exhibits a fitness defect during colonization [63]. The fitness defect observed was suggested to be owed to resistance of this strain to phage lysis, due to alterations in the cell wall that lead to tolerance to the phage, and a negative effect on fitness. While the mechanism of release and binding of PblB has not been demonstrated in *S. pneumoniae*, it could rely on phage-mediated cell lysis, as described in *S. mitis*. Hence, modifications in the cell wall composition that affect lysis could also limit the release of PblB. Interestingly, lysogeny with MM1-1998 was shown to improve adherence to nasopharyngeal cells [80]. MM1-1998 is nearly identical to phage MM1, and does not encode homologs of PblA nor PblB [20,26,80]. These findings suggest that other phage-encoded factors could contribute to adhesion.

Contradictory findings on the role of phages in colonization and disease are perhaps not unforeseen given the wide genetic diversity of pneumococcal phages and the extraordinary plasticity of the pneumococcal genome, where the core genome constitutes merely a quarter of the pneumococcal pangenome [81]. It is thus likely that more factors than the simple presence of a phage, or a specific phage-encoded feature such as PblB, are at play. This is illustrated by the fact that lysogeny with phage MM1-1998 does not lead to the same phenotypes in strains of different serotypes and genetic backgrounds [80].

## 6. Phages, Biofilms, and Natural Competence for Transformation

Interactions between phages and their hosts are complex, and benefits for the pneumococcus derived from lysogeny could likely be diverse. For example, pneumococcal phages have been associated with antibiotic resistance, through co-integrate formation with the Tn*916* integrating conjugative element [20], or by inducing cell wall modifications that increase resistance to penicillin-mediated cell lysis [63]. A lysogen of pneumococcal phage SV-1 has also been shown to exhibit a faster and more massive biofilm development than the corresponding non-lysogenic strain, due to spontaneous prophage activation. The concomitant increase in cell lysis is dependent on the action of the phage and the host lytic amidases, and leads to an increase in release of extracellular DNA, an important component of biofilms [71]. Biofilms are an appropriate environment for genetic exchange through natural transformation, and cell-to-cell contact was found to promote the import and recombination of large DNA sequences in *S. pneumoniae* [82]. It appears likely that the extracellular DNA released by phage induction would also contribute to promote genomic plasticity through natural transformation.

In stark contrast, phages of group 2b have been shown to inhibit natural transformation through integration within the coding sequence of *comGC* (Figure 2a) [53]. Inhibition of natural transformation is thought to be a protective mechanism adopted by certain integrative mobile genetic elements to prevent being deleted from the chromosome following uptake of DNA from a non-lysogenic strain by their host [22]. While this is thought to be a phage protective mechanism [22], it would also have dramatic consequences for the evolutionary potential of such lysogens. Indeed, interruption of *comGC* by prophages has been shown to be associated with a decrease in carriage duration, possibly by neutralizing the ability of the lysogens to eliminate harmful mutations [53,83]. Interestingly, phages and competence are further intertwined, as activation of RecA during transformation also leads to phage induction, even in absence of DNA-damaging agents [33]. Ultimately, phage contribution to genome evolution, biofilm formation, and antibiotic resistance would be expected to enhance fitness in certain environments, which, in turn, could influence pneumococcal pathogenesis.

## 7. Phage-Related Chromosomal Islands

Genomic analyses have confirmed that a significant proportion of pneumococcal isolates carry phage remnants and phage-related chromosomal islands (PRCIs) [20,21,72]. Because they lack genes encoding the structural components of phage particles, PRCIs—also called PICIs (phage-inducible chromosomal islands)—are unable to disseminate by themselves and require the presence of a helper phage to mobilize their genetic material. PRCIs have been extensively studied in *Staphylococcus aureus*, and the *S. aureus* pathogenicity islands (SaPIs) are the prototypical members of this family of mobilizable genetic elements. PRCIs harbor specific genetic modules involved in integration/excision, regulation, autonomous replication, and helper-phage exploitation [84]. The accessory gene content carried by PRCIs is diverse, encompassing virulence genes such as the toxic shock syndrome toxin 1 (TSST-1) encoded by SaPIs, biofilm-inducing proteins, and antibiotic resistance genes (reviewed in [84]). PRCIs described in *S. pneumoniae* distinguish themselves from phage remnants and complete phages by a distinctive gene content and different chromosomal integration sites [21,72,85]. Unlike temperate phages, SaPI excision and replication is not triggered directly by DNA-damaging agents, but rather indirectly via de-repression mediated by a co-resident helper phage following its induction [84,86]. Pneumococcal PRCI genes were shown to be expressed in the same conditions as co-resident prophage genes [72], however, whether this induction also depends on phage-encoded features is not known. The mechanistic details of the dissemination and overall contribution to horizontal gene transfer of PRCIs have not been studied in the pneumococcus. Due to the propensity of *Spn* for natural transformation, it is possible that transformation, followed by RecA-mediated homologous recombination, contributes in part to PRCIs mobilization. The knowledge on the accessory functions harbored by *Spn* PRCIs is scarce, but SpnSP38 was recently shown to enhance survival in serum, and thus increase the bacterial load in the blood in a sepsis infection model [72].

## 8. Perspectives

Phages are increasingly recognized as important players in the human microbiome, and they appear to be a significant component of the respiratory virome [87,88,89,90]. The contribution of the microbiome in human health and physiology is undeniable, and while understudied in comparison with the gut microbiota, the respiratory microbiome is emerging as a major player in respiratory health (reviewed in [91]). Bacterial species closely related to *S. pneumoniae*, such as *S. mitis*, *Streptococcus oralis,* and *Streptococcus pseudopneumoniae,* are common constituents of the oral and respiratory tract flora, and these species are also known to carry temperate phages [72,92]. There is evidence for common genetic content between these phages, such as Int2a, which is found in *S. pneumoniae* and *S. pseudopneumoniae* phages, and the homologs of *pblA* and *pblB*, which are present in both *S. pneumoniae* and *S. mitis* phages [26,39,92]. A broader genomic study has found that similar phages are present in distinct streptococcal species, even those which are not the most closely related [72]. The lack of CRISPR systems in *S. pneumoniae,* combined with the identification of CRISPR spacers homologous to *S. pneumoniae* phages sequences within salivary samples, suggests that these phages have a host range that extends beyond the pneumococcus [93,94,95]. How cross-species transmission and phage immunity mechanisms contribute to shaping the host respiratory tract microbiome landscape is still unknown. Given the interconnection between temperate phage induction, release of extracellular DNA, and the ability of streptococci of the mitis group for natural transformation, phages could play an even greater role in the genetic diversity of these species. This could be particularly important in the context of antibiotic use, where some antibiotics utilized in the treatment of respiratory tract infections, such as fluoroquinolones, lead to induction of both competence and temperate phages [31,34,35]. It is likely that interactions between phages and susceptible hosts, as well as between lysogens and non-lysogens of the same, or different, species, also contributes to shape the structure and composition of the respiratory microbiome. Phages can, for example, inadvertently partake in the ferocious rivalry between bacteria competing for the same ecological niche. One striking case is the unique strategy adopted by *S. pneumoniae* to compete with *S. aureus*, mediated by the production of sublethal amounts of hydrogen peroxide [96,97,98]. *S. aureus* isolates are usually lysogenic and, while *Spn* is itself resistant to its effects, the H_2_O_2_ it produces triggers the induction of the SOS response in *S. aureus* and the concomitant initiation of the lytic cycle of its phages [98].

While extremely relevant, the broad picture of phage distribution obtained by large-scale genomic and metagenomic analyses must also be complemented with more detailed mechanistic studies, since assessing functionality of phages is critical to fully understand the impact of their distribution. The functions encoded by the majority of pneumococcal phage genes remain unknown, both in the context of the phage life cycle and for their impact on host bacterial gene expression. Many questions indeed remain open today in terms of phage biology in the pneumococcus. The identification of phage anti-receptors and their counterpart receptors on the host surface is but one of them. Harriet Bernheimer observed already in 1979 that the pattern of pneumococcal susceptibility to infection by a certain set of phages was suggestive of the lack of the appropriate receptor on some isolates [17]. More than four decades later, this observation remains unverified. Uncovering which genetic features dictate specific phage–host associations will also cast light onto the host range and the impact of temperate phages in the context of globally-relevant pneumococcal lineages. These interactions could very well be through genetic cross-talk between chromosomal and phage genes. Indeed, studies in *E. coli* have shown that phage-encoded transcriptional regulators can modulate the expression of chromosomal and pathogenicity island genes, impacting both fitness and virulence of the lysogens [99,100]. The existence of similar interactions between the pneumococcus and its temperate phages is unknown, but this is plausible, given the numerous putative transcriptional regulators and DNA binding proteins annotated in pneumococcal phage genomes. The large number of phage genes encoding hypothetical proteins highlights how little we yet know about temperate pneumococcal phages and their influence on pneumococcal biology.

## Appendix A

**Table A1:** Accession Numbers of Phages and Pneumococcal Genome Sequences Used in Figure 2.

Phage Name	Phage Accession Number	Genome Accession Number
IPP12	KY065454.1	NC_012467.1
IPP39	KY065479.1	AGPM01000004.1
phiSpn_200/IPP42	KY065482.1	NC_014494.1
IPP43	KY065483.1	NZ_UHGQ01000001.1
IPP45	KY065485.1	CP002176.1
IPP46	KY065486.1	CP002176.1
IPP48	KY065487.1	CP003357.2
IPP51	KY065489.1	AGPN01000004.1
IPP53	KY065491.1	AGOL01000005.1
phiOXC141/IPP57	KY065494.1	NC_017592.1
IPP58	KY065495.1	AGQI01000002.1
IPP60	KY065496.1	NZ_FYOS01000005.1
IPP67	KY065503.1	NZ_ABWQ01000001.1
MM1	NC_003050.2	FM211187.1
phi34117	FR671407.1	Not available
phi8140	FR671410.1	Not available
phiNTCC11888	Used genome	NZ_UHFY01000001.1
phiNTCC13735	Used genome	NZ_UHGD01000001.1
phiSMRU1311	Used genome	NZ_CRHW01000001.1
phiSMRU2522	Used genome	NZ_CLJV01000005.1
phiSMRU3096	Used genome	NZ_CMLU01000003.1
phiSPNH_2	Used genome	CP000936.1
phiUoS0268	Used genome	NZ_FYHY01000007.1
phiUoS4388	Used genome	NZ_FYVE01000006.1
SPN195_1	Used genome	ABGE01000002.1
SPN195_2	Used genome	ABGE01000013.1
